# Computational Identification of Active Drug Metabolites for Human Protein Targets

**DOI:** 10.1021/acs.molpharmaceut.6c00258

**Published:** 2026-06-18

**Authors:** Sofia Larsson, Rocío Mercado, Susanne Winiwarter, Filip Miljković

**Affiliations:** † Department of Computer Science and Engineering, 11248 Chalmers University of Technology and University of Gothenburg, Chalmersplatsen 1, 412 96 Gothenburg, Sweden; ‡ Drug Metabolism and Pharmacokinetics, Research and Early Development, Cardiovascular, Renal and Metabolism (CVRM), BioPharmaceuticals R&D, AstraZeneca, Pepparedsleden 1, 431 83 Mölndal, Sweden; § Biopharma Chemistry, Discovery Sciences, 468087 BioPharmaceuticals R&D, AstraZeneca, Pepparedsleden 1, 431 83 Mölndal, Sweden

**Keywords:** drug discovery, drug metabolism, compound bioactivity, pharmacokinetics, data mining

## Abstract

Understanding drug metabolism helps mitigate toxicity risks and anticipate pharmacological effects beyond the parent compound. Here, we explore high-confidence *in vitro* human bioactivity annotations from the public domain associated with a curated data set of drug-metabolite pairs. Our findings show that 26% of all drug-metabolite-target combinations contain metabolites with retained or increased bioactivity relative to their parent drugs, showing a high proportion of active metabolites with potencies below 100 nM. Moreover, these active metabolites display similar predicted physicochemical and ADME properties to their parent drugs, indicating that their biological activity may be preserved *in vivo*. Two key features of the identified active metabolites are noted: (1) the metabolite is more likely to be formed via an O-demethylation or an N-demethylation at a tertiary nitrogen, and (2) these drug-metabolite pairs are frequently active against the membrane receptor target class. To support further research in this area, we make our data compilation, including pairs with identified active metabolites and recorded bioactivity data, openly available.

## Graphical abstract

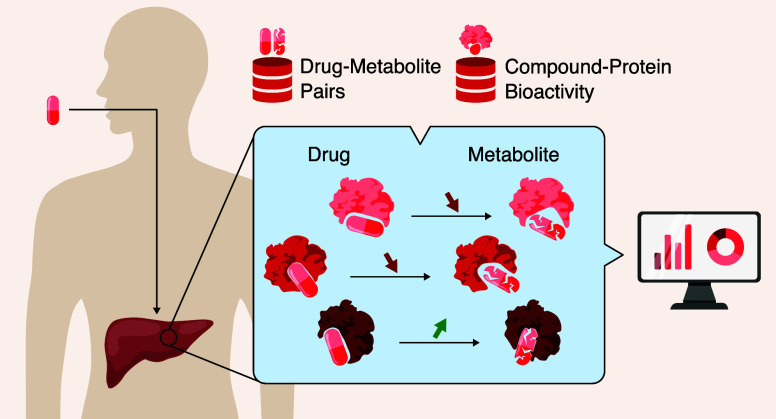

## Introduction

Drug metabolism is the body’s defense mechanism aimed at reducing the potential effects of a foreign compound (i.e., a xenobiotic) by enabling rapid elimination from the exposed organism and/or inactivation of the compound in question. Metabolism reactions such as hydroxylations, oxidations, dealkylations, and conjugations are most common^1^ and serve two goals: (1) increased hydrophilicity of the chemical matter enables fast elimination via the urine^2^ and generally leads to lower pharmacological activity,^3^ and (2) metabolite reactions, such as conjugation, may in addition target the resulting metabolite for transporter interactions and enable fast active excretion via the bile.^4^

In drug discovery, it is critical to understand these mechanisms early in the process to guide compound optimization toward clinical candidates, counteract metabolism, and anticipate when active or reactive/potentially toxic metabolites may arise. Preliminary evaluation of the compound’s rate of metabolism is typically obtained through the use of *in vitro* (tier 1) assays during the design-make-test-analyze (DMTA) cycle,^5^ −^6,7^ whereas the more elaborate identification of metabolites is usually performed on specific compounds only. Both qualitative and quantitative information describing compound metabolism is fed into the next design round to develop novel molecules.

Recent advances in computational method development, as well as the growth of sufficiently large, representative, and well-curated data, have allowed for the generation of highly predictive *in silico* models addressing the rate of metabolism estimation.^8–10^ In that regard, the rate of metabolism is a “simple” number that needs to be correlated to compound structure. In contrast, site-of-metabolism and metabolite prediction models operate in low data regimes and require different, more specialized data curation protocols, including a way to properly describe the available experimental data for computational methods to efficiently parse and learn from.^11,12^ Further complexity with such experiments and data arises when an exact metabolite cannot be identified, necessitating that the entire region of a molecule rather than a single atom or a functional group is highlighted for metabolic lability (e.g., an entire benzene ring instead of a particular position).^1^

Additionally, depending on the purpose of the metabolite identification experiment, sometimes only a few major metabolites are reported, which in turn constrains the completeness of *in silico* models derived from such data. Going beyond predictive *in silico* approaches, a systematic evaluation of publicly available bioactivity records associated with metabolites and their parent drugs may reveal how these metabolic transformations relate to the observed differences in compound bioactivity at large. This would also consequently allow for the identification of active drug metabolites that may, in some cases, pose safety- and toxicity-related risks in humans.

We recently curated a data set of drug-metabolite pairs based on publicly available data to develop a transformer-based language model for the prediction of drug metabolites.^12^ Here, we further expand on this data set to assign high-confidence bioactivity annotations associated with human protein targets to drugs and their metabolites, using two large public data repositories, namely ChEMBL^13^ and BindingDB.^14^ We apply stringent data curation criteria to obtain drug-metabolite-target combinations to which reliable activity measurements are assigned and directly compared. Then, we distinguish and focus on drug-metabolite-target combinations containing metabolites with either retained or increased bioactivities (i.e., “active metabolites”) from other such combinations. We compare calculated and predicted physicochemical, and ADME (absorption, distribution, metabolism, and excretion) properties of drugs and their metabolites and discuss their lipophilicity and permeability profiles. Finally, we explore which metabolic transformations are likely to produce active metabolites and which protein target classes they often target.

## Methods and Materials

### Data Sets

We used a previously curated data set of drugs and their metabolites in humans, named LAGOM^12^ as the basis for this work. It was compiled by extracting drug-metabolite pairs from DrugBank^15^ and MetXBioDB.^16^ During the data curation, compounds containing elements other than C, O, N, Cl, F, S, P, Br, and I were discarded. In addition, the Tanimoto similarity (1024-bit Morgan fingerprints, radius 2) between the parent and the metabolite was required to be > 0.2. The data set was extended with the GLORYx test set,^17^ containing 136 drug-metabolite pairs related to the top-selling drugs of 2018. The extended LAGOM data set contains 4191 unique drug-metabolite pairs, including 5322 unique compounds represented as SMILES strings (Figure 1a).

**Figure 1:**
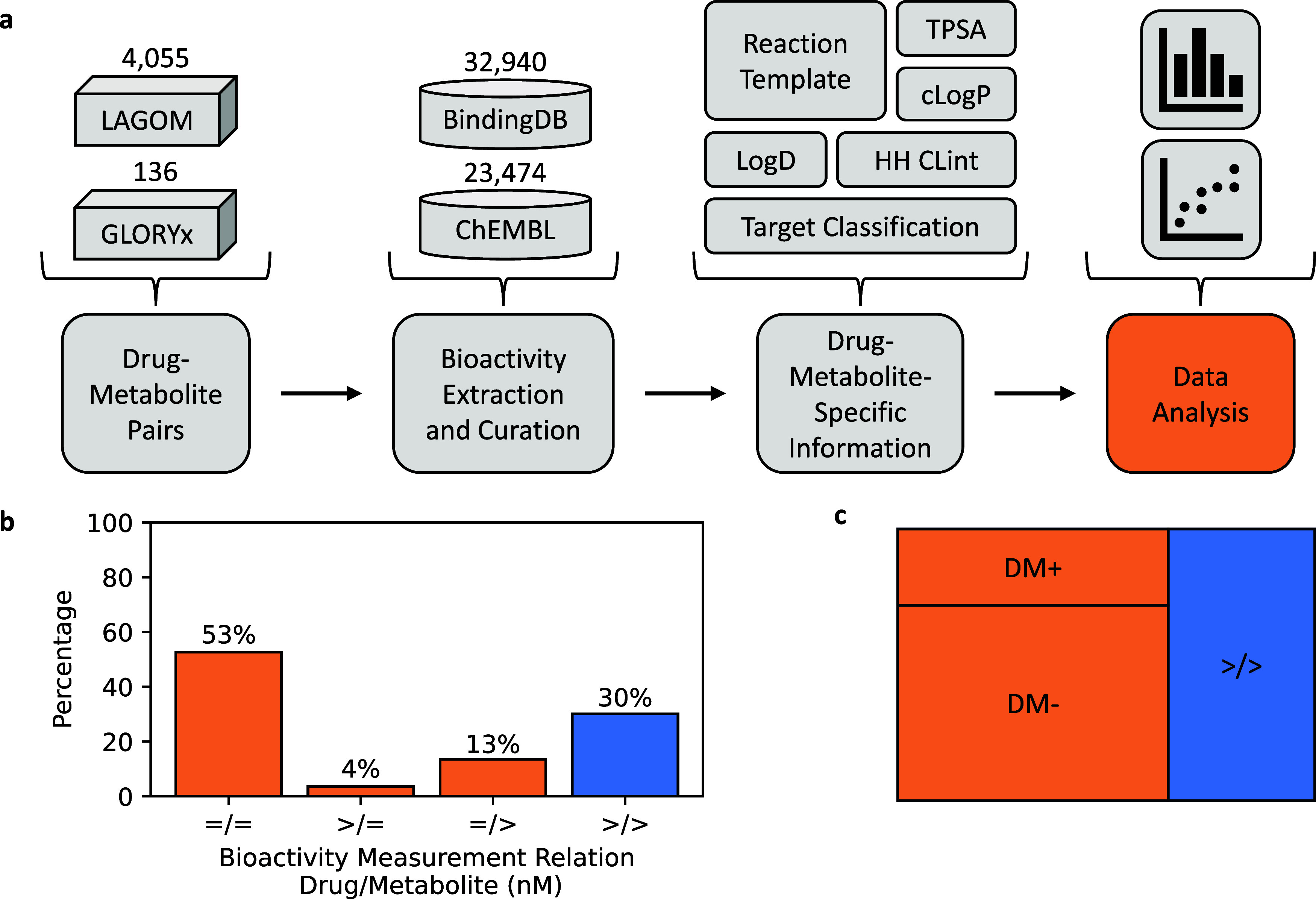
Overview of the data set extraction process. (a) Pipeline of the project, beginning with the collection of drug-metabolite pairs, extraction of the corresponding bioactivity data and their curation, addition of drug-metabolite-specific information, and ensuing data analysis. (b) Histogram of different bioactivity measurement relations (given in nM values) found in the final data set (849 reaction-target pairs) after curation, where “=/=”: both the drug and its metabolite have exact measurements; “>/=”: drug has “>” (greater than) relation and its metabolite an exact measurement; “=/>”: drug has an exact measurement and its metabolite has “>”; “>/>”: both the drug and the metabolite have “>” relation. (c) Data subsets of interest for data analysis. The full box represents the data set from (b), showing that the subsets of interest exclude “>/>” measurements. The DM+ subset contains reaction-target pairs where the metabolite has retained or increased bioactivity (i.e., active metabolite) as per defined constraints. The DM– subset contains reaction-target pairs, where the metabolite is considered inactive or has lower bioactivity than the parent drug.

### Data Extraction and Curation

We extracted biological assay information associated with the curated drugs and their metabolites in the extended LAGOM data set from CHEMBL 36 ^13,18^ and BindingDB (release 2025–09–01),^14^ two major repositories of compound bioactivity data, using standardized SMILES matching.^19,20^ This is visualized as the second step in Figure 1a. To ensure the highest assay data quality, we defined a set of constraints inspired by the curation done by Xerxa et al.^21^ First, bioactivity measurements from ChEMBL were required to be reported in:

- molar units (e.g., pM, nM, μM, mM, or M),
- IC_50_, K_d_, or K_i_ as measurement type,
- *Homo sapiens* single-protein targets,
- direct-binding assays (“D”) with a confidence score of 9 (highest),
- and cell-free assays (no cell type reported in the assay; pure protein target).

Similarly, BindingDB bioactivity measurements were required to be reported in:

- IC_50_, K_d_, or K_i_ as measurement types,
- and single-chain *H. sapiens* protein targets.

We mapped each protein target from ChEMBL to its unique UniProt identifier (UniProt ID). Protein targets from BindingDB were already associated with their UniProt IDs. This resulted in a total of 15,572 unique compound-target pairs consisting of 1287 unique compound SMILES following the database merge.

Each compound bioactivity measurement was associated with a concentration value and a defined activity relation (e.g., “=”, “>” and “<”) in both databases. These relations come from the original data sources and in the case of measurements with “>” and “<” signs they reflect either the measurement detection limits or inability to confidently capture exact experimental values. When an exact value is determined, the “=” relation is used. When no effect is observed up to the highest tested concentration, the “>” relation (e.g., “>1,000 nM”) is used. Additionally, some activity relations appear as “≥”, “≤” and “∼” in the data. To handle these inconsistencies, we standardized these relations such that the activity relations with the same direction (“≥” and “>”, and “≤” and “<”) retain a single directed relation (“>” and “<”, respectively). Moreover, we removed the instances with “∼” (only four cases). We also discarded all data points with a “<” relation (462 data points), motivated by the difficulty of interpreting bioactivities of compounds in ranges where exact measurements are expected (e.g., low nanomolar inhibitors). Next, we discarded any data point with the “>” relation and a reported bioactivity below 10,000 nM. This was done based on the observation that “>10,000 nM” measurements are typically reported as the upper experimental limit of many bioactivity assays in ChEMBL. Furthermore, any data point with the “=” relation that had a reported bioactivity above 10,000 nM was discarded given that beyond this range the exact compound bioactivity is less precise. Finally, compound-target pairs containing both exact and “>” measurements were evaluated for agreement. If the exact measurement was greater than the value(s) associated with the “>” measurement, then we kept only the exact measurement, else both were discarded. This ensured that for each compound-target pair associated with one of the activity measurement types (IC_50_, K_d_, or K_i_) only a single measurement relation was kept to record the activity.

Next, for compound-target pairs with multiple “>” measurements (in original units) within a particular measurement type (i.e., IC_50_, K_d_, or K_i_), the lowest reported value for the compound-target pair was recorded. For instance, for a compound-target pair with “>15,000 nM” and “>13,000 nM”, the value of “>13,000 nM” was kept. The single retained value was then converted to the corresponding negative base 10 logarithm of the value represented in molar units (i.e., pIC_50_, p*K*_d_, or p*K*_i_). For compound-target pairs containing exact bioactivity measurements (i.e., “=”), the values were averaged for each recorded measurement type in logarithmic units (i.e., pIC_50_, p*K*_d_, or p*K*_i_) and discarded if their standard deviations were ≥ 1.

This curation resulted in a total of 878 drug-metabolite-target combinations (hereafter referred to as *reaction-target pairs*) across all three measurement types (pIC_50_, p*K*_d_, or p*K*_i_), consisting of 236 drug-metabolite pairs, 372 unique compounds, and 419 protein targets. The final curation step was to retain a single measurement type per reaction-target pair using the following priority order: K_i_ > IC_50_ > K_d_. The order was established to give preference to: (1) inhibition measurements (K_i_ and IC_50_; note that K_i_ can also reflect binding affinity in the case of agonists^22^) over the binding measurements (K_d_), as we are more interested in the functional response than binding affinity alone, and (2) assay-independent values (K_i_) over assay-dependent values (IC_50_). Following this, 849 reaction-target pairs across all three measurements were obtained. The distribution of bioactivity measurement relations in the data set can be seen in Figure 1b.

### Drug-Metabolite-Specific Information

The data set was further enhanced with calculated or predicted physicochemical and ADME properties of the drugs and their corresponding metabolites, as well as the protein target data and reaction classes (Figure 1a).

#### Physicochemical and ADME Descriptors.

Using RDKit,^19^ we calculated **cLogP** (calculated LogP) and **TPSA** (topological polar surface area) for all drugs and metabolites. cLogP is the calculated partition coefficient of a molecule between the octanol and the water phases, accounting for its lipophilicity. TPSA is a two-dimensional (2D) molecular descriptor calculating the surface sum over all polar atoms (primarily oxygen and nitrogen, with attached hydrogens) to estimate drug permeability, absorption, and bioavailability. Both cLogP and TPSA are properties relevant for assessing compound lipophilicity and permeability through cell membranes. In addition, **LogD**_7.4_ and **HH CLint** (human hepatic intrinsic clearance) values were predicted for each drug and metabolite. LogD_7.4_ is the distribution coefficient of a molecule between the octanol and the water phases at pH 7.4, accounting for both neutral and ionized forms of molecules, and thus more physiologically relevant than cLogP for compounds that contain acidic and/or basic functional groups. LogD_7.4_ values were obtained from the publicly available model reported in Fu et al.^23^ HH CLint is the property that describes the metabolic stability of compounds in human hepatocytes, accounting for both Phase I and Phase II metabolic enzyme transformations. HH CLint values were predicted for each drug and metabolite using an internal AstraZeneca model as described in Gawehn et al.^10^

#### Reaction Classes

In order to group different drug-metabolite transformations into common reaction mechanisms, SMARTS reaction templates were extracted from the drug-metabolite pairs. This was done using rxnmapper^24^ for atom mapping and, thereafter, reaction_utils (rxnutils)^25^ for extracting the reaction templates as SMARTS. The parameter radius for extracting the templates, which corresponds to the number of atoms away from the reaction center to be considered, was set to zero to obtain the most general templates. We obtained 121 unique reaction templates from our 236 unique drug-metabolite pairs in the data set.

#### Target Classes

For each protein target, we obtained additional target classification information from ChEMBL to further examine drug-metabolite transformations at different protein classification levels. The classifications are hierarchical, spanning from the individual protein targets at leaf nodes to major protein classes at the roots. For example, histamine receptor and dopamine receptor are protein targets that both belong to the “monoamine receptor” target class, just like endothelial differentiation gene receptor and prostanoid receptor are members of the “lipid-like ligand receptor” target class; furthermore, both of these families belong to the “membrane receptor” target class.

### Bioactivity Comparison

We then compared the bioactivities of drugs to those of their metabolites. Reaction-target pairs where both the drug and its metabolite had out of bound (“>/>”) measurements were removed from the analysis, since no insights could be derived from such pairs. This resulted in 593 reaction-target pairs remaining for analysis. Pairs where the bioactivity of the metabolite was higher or similar to its drug (but not weaker than 0.5 logarithmic units) and had pIC_50_, p*K*_d_, or p*K*_i_ of at least 7 (≤100 nM) were designated as the “DM+” subset and treated separately. The remaining combinations constituted the “DM–” subset. A visualization of the two subsets is shown in Figure 1c.

As the retention or increase of metabolite bioactivity in the DM+ pairs could be attributed to prodrug-to-drug conversion (i.e., a drug-metabolite pair would in this case constitute a prodrug-drug pair) we further investigated which of the drugs associated with these pairs were defined as prodrugs in ChEMBL (“Prodrug Info” tab under each compound). For pairs with confirmed prodrugs (“Dosed Ingredient”), we noted whether their metabolites were designated active metabolites (“Active Ingredient”) in ChEMBL (defined under the “Prodrug Info” tab). For these pairs, if either a prodrug or its active metabolite had a defined mechanism of action target (“Drug Mechanisms” tab under each compound) that corresponded to the recorded protein target of the reaction-target pair, then this pair was considered to be previously known.

## Results and Discussion

### Bioactivity Analysis

Figure 2 shows the distributions of exact bioactivity measurements (“=/=”) for drug-metabolite pairs (447 reaction-target pairs) across all three activity measurement types in the data set (p*K*_i_, pIC_50_, and p*K*_d_). Using the defined constraints specified in the Bioactivity Comparison section, 34% of drug-metabolite pairs contained metabolites with retained or increased bioactivity measurements compared to their drugs, with p*K*_i_ (Figure 2a) having the highest proportion of such pairs. If the entire data set of reaction-target pairs (593 reaction-target pairs) with bioactivity measurements of interest is considered, 155 (26%) of all reaction-target pairs contained metabolites with retained or increased bioactivity (the DM+ subset). This demonstrates that active drug metabolites, i.e., products of drug metabolism with protein bioactivity consistent with or higher than the corresponding drug molecule, are frequently observed in public bioactivity data repositories. Moreover, for 35% of pairs, the associated metabolite was reported with bioactivity better than or equal to 100 nM (p*K*_i_, pIC_50_, and p*K*_d_ ≥ 7), showing that highly potent metabolites, irrespective of the bioactivity reported for their parent drugs, are commonly observed in the public data sources. These findings are surprising, given the general view in drug discovery that drug metabolites are typically inferior compared to their parent drugs. An exemption from this rule are prodrugs: weakly active or inactive chemical compounds that rapidly metabolize *in vivo* into highly potent molecules active against intended protein targets. Thus, we surveyed which of these DM+ reaction-target pairs contained a prodrug as per ChEMBL definition. We identified nine prodrugs that comprised 14 reaction-target pairs (9%) where the ChEMBL mechanism of action target matched the recorded protein target of the pair. This indicates that only a small fraction of the DM+ subset was previously known to yield active metabolites via the prodrug mechanism, thus rendering the majority of DM+ pairs novel.

**Figure 2:**
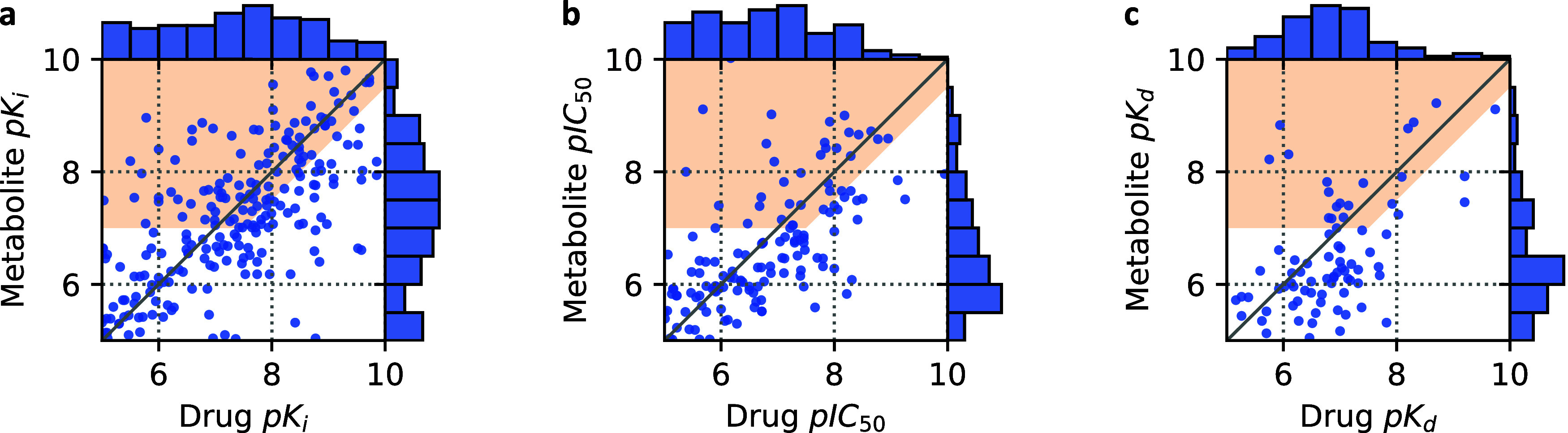
Distribution of exact (“=/=”) bioactivity measurements in the data set (447 reaction-target pairs) for different measurement types. The orange area indicates zones where metabolites have retained or increased bioactivity in comparison to the parent compounds (*y* = 7 and *y* = *x*–0.5), 34% of the data, with the exact breakdown as follows: (a) p*K*_i_ (97/229), (b) pIC_50_ (36/140), and (c) p*K*_d_ (18/78).

### Comparison of DM+ and DM–

In Figure 3 we compare the physicochemical and ADME properties between drugs and their corresponding metabolites for both the DM+ and DM– subsets. Overall, both the DM+ and DM– subsets show similar overlapping regression lines for all properties. Both Figure 3a and 3c show similar lipophilicity trends across both subsets where the metabolites are slightly less lipophilic compared to the corresponding drugs. This is expected given that metabolic transformations typically produce more hydrophilic metabolites, which improves their excretion. Figure 3b shows the tendency of metabolites to have a slightly higher TPSA than the respective drugs (on average 7.3 Å^2^ and 11.4 Å^2^ greater for the DM+ and DM– respectively). This is also reasonable since higher TPSA indicates a more polar compound. In Figure 3d, the overall predicted HH CLint trend shows that metabolites are metabolically more stable (low HH CLint) compared to their drugs. However, these observations show lesser confidence given the widespread of data points in the scatter plot. Nevertheless, this aligns with the expectation that metabolites are typically more metabolically stable compared to their parent drugs due to the presence of hydrophilic functional groups.

**Figure 3:**
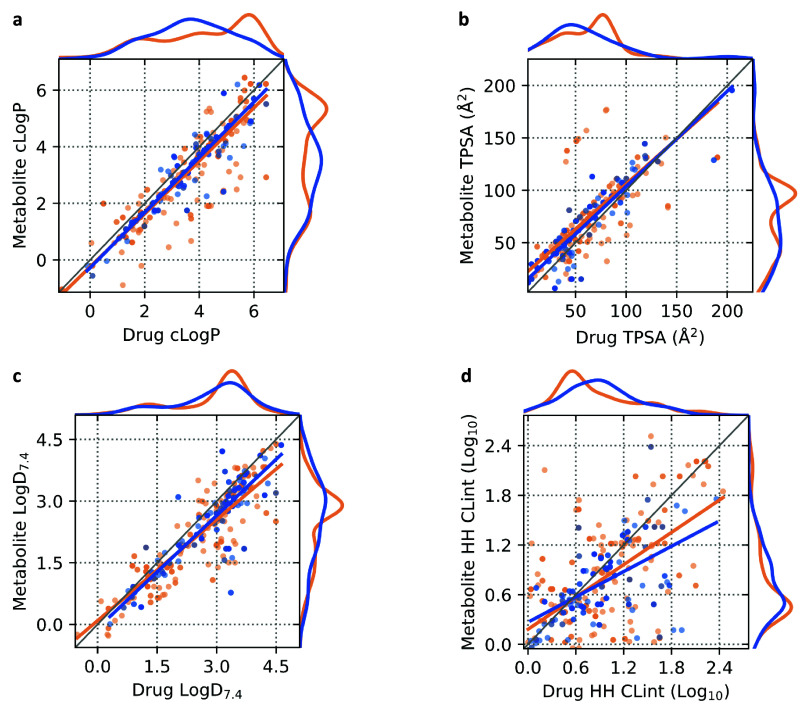
Distributions of physicochemical and ADME properties for reaction-target pairs in DM+ (blue) and in DM– (orange), with corresponding regression lines. (a) Calculated logarithmic octanol–water partition coefficient (cLogP; unitless). (b) Calculated topological polar surface area (TPSA; Å^2^). (c) Predicted logarithmic distribution coefficient (LogD_7.4_; unitless). (d) Predicted logarithmic human hepatocyte intrinsic clearance (HH CLint; μL/min/million cells in the original unit). There is no significant difference between the DM+ and DM– sets in terms of physicochemical properties. The regression lines are in agreement with expected drug-metabolite behavior.

Table 1 shows the top ten drug-metabolite reaction groups found in the DM+ subset and provides their frequency and percentage in both the DM+ and the DM– subsets. Ratios for reaction groups in the DM+ subset that are present in greater proportion than across all pairs (>26.1%) are highlighted. In other words, these reaction groups preferentially feature reaction-target pairs with active metabolites compared to pairs where the metabolites were either less active than their parent drugs or entirely inactive. The top two most frequent reaction groups, namely O-demethylation and N-demethylation (tert. N), were found to be overrepresented in the DM+ subset, where nearly an equal number of instances were found both in the DM+ and the DM– subsets (yet, the DM+ subset is three times smaller than the DM– subset). Other reaction groups such as deoxygenation (sulfoxide), O-dealkylation, conjugation (diverse), hydroxylation (aliphatic chain), and oxidation (ketone) are present in even greater proportion of pairs but they are too few to draw any clear conclusions. On the other hand, hydroxylation (aliphatic ring) and N-demethylation (sec. N) were less frequently represented in the DM+ subset, indicating that such conversions are less likely to lead to the formation of active metabolites.

**Table 1:** Top Ten Reaction Groups in the DM+ Subset^a^,^b^

reaction group	in DM+		in DM–	
	#	%	#	%
data set	155	26.1	438	73.9
O-demethylation^b^	26	**48.1**	28	51.9
N-demethylation (tert. N)^b^	22	**43.1**	29	56.9
hydroxylation (aliphatic ring)^b^	19	11.9	140	88.1
hydroxylation (aromatic)^b^	6	25.0	18	75.0
deoxygenation (sulfoxide)	6	**75.0**	2	25.0
O-dealkylation	4	**100.0**	0	0.0
conjugation (diverse)	4	**44.4**	5	55.6
hydroxylation (aliphatic chain)	4	**80.0**	1	20.0
N-demethylation (sec. N)^b^	4	12.5	28	87.5
oxidation (ketone)	4	**40.0**	6	60.0

a Note that the same reaction template can occur several times for the same drug-metabolite pair due to their bioactivity measurements recorded for different targets. The ratio (%) is for the specific reaction group found in the subset. Ratios for the reaction groups where their proportion is greater than that reported for the entire DM+ subset are highlighted. Corresponding reaction template SMARTS are found in Table S1 in the Supporting Information

b Top five reaction groups in DM– subset.

Figure 4 shows the proportion of reaction-target pairs for each recorded target class at the highest protein classification level, given for both DM+ (Figure 4a) and DM– (Figure 4b) subsets, with a further breakdown of the enzyme class into individual families. As shown in the figure, membrane receptors dominate the DM+ subset (53%) compared to DM– where they only amount to a quarter of the reported pairs (23%). On the other hand, enzymes are by far more frequently recorded in the DM– subset (52%) compared to the DM+ subset (29%). Other target classes are equally represented in both subsets, with the exception of secreted targets that are highlighted in the DM+ subset, whereas their presence in the DM– subset is only minor and aggregated with other less frequently reported classes (“Other”). Further breakdown of the enzyme class reveals that kinases are somewhat more represented in the DM– subset (64% compared to 56% in the DM+ subset), whereas lyases are slightly more present in the DM+ subset (16% compared to 14% in the DM– subset). There were also enzyme families that were recorded in one group but not in the other, and *vice versa*. Here, the phosphodiesterase family is exclusively found in the DM– subset, while the ligase family is reported only in the DM+ subset. Other target classes were recorded in both subsets, with no major differences noted.

**Figure 4:**
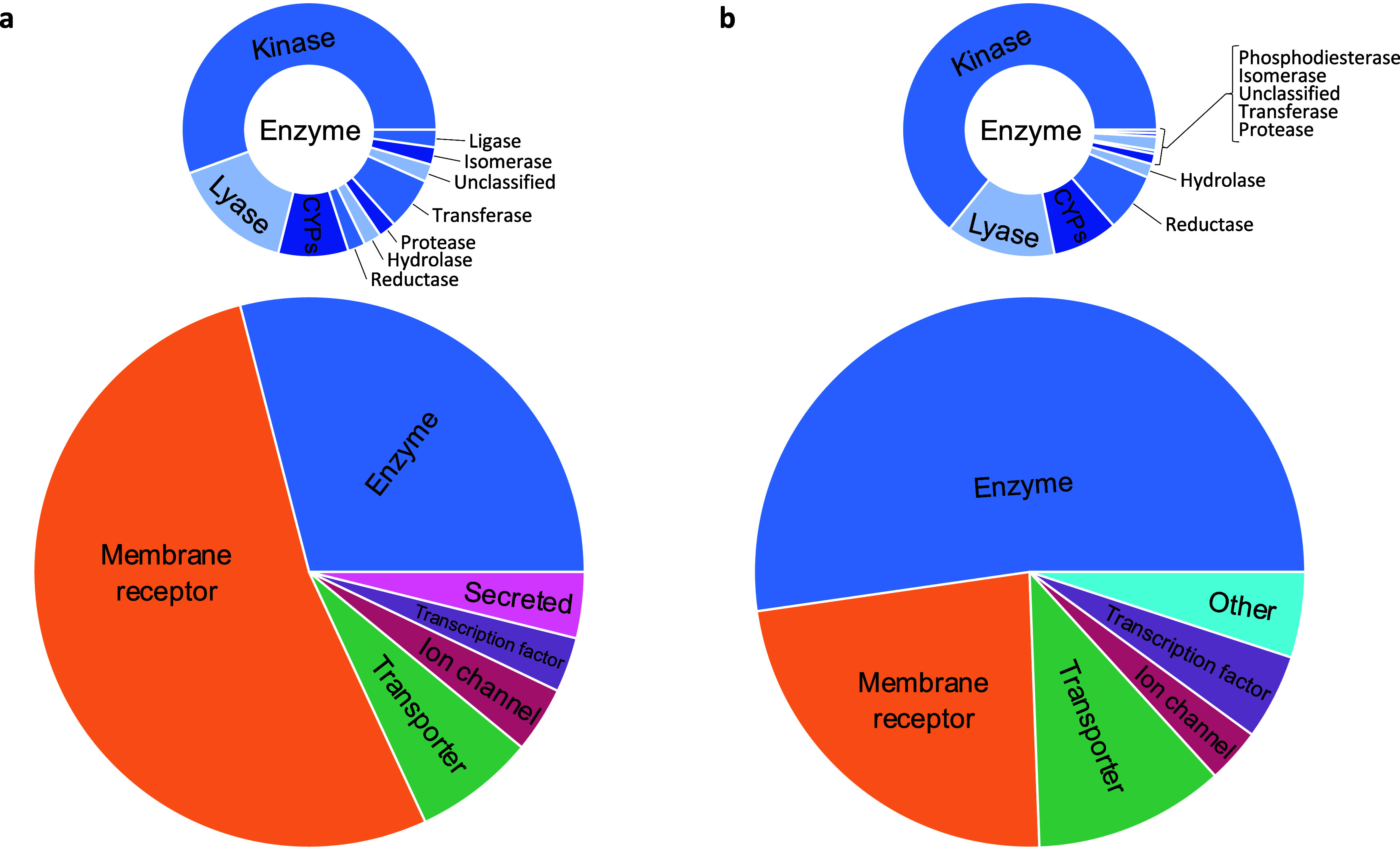
Distribution of targets in the two subsets. Membrane receptors dominate the DM+ subset whereas enzymes form the majority in the DM– subset. (a) DM+ subset of 155 reaction-target pairs. (b) DM– subset of 438 reaction-target pairs. In (b), “Other” indicates a merging of classes in the DM– subset with count < 10.

### Exemplary Drug-Metabolite Pairs

Next, we looked at drug-metabolite pairs from both subsets involving different cases where metabolites with increased or decreased bioactivity were observed (Figure 5). Note that stereochemistry was removed during the curation process of this data set. The first example, tamoxifen, is a known estrogen receptor α inhibitor used in the treatment of estrogen receptor positive breast cancers^26^ (top left). Its active metabolite 4-hydroxytamoxifen^27^ (top right) displays an even greater inhibitory activity against the same target, according to the curated bioactivity data from public data sources (pIC_50_ = 8.50 compared to pIC_50_ = 6.80 for tamoxifen). This example demonstrates how a simple, common metabolic transformation, such as phenyl hydroxylation in the para position, can lead to the formation of an active metabolite with a significantly greater activity compared to its parent drug, here resulting in nearly a 100-fold potency gain. This discovery has led metabolite 4-hydroxytamoxifen, also known as afimoxifene, to be clinically investigated as a topical treatment for cyclical mastalgia in premenopausal women.^28^ Afimoxifen exemplifies an active drug metabolite that has been repurposed as a distinct clinical entity independent of its parent drug. The next example shows delamanid^29^ (second left), an antituberculosis agent, and its metabolite DM-6705 (second right) that is formed via hydrolytic cleavage.^30^ While an antibiotic, delamanid is not only required to exhibit a strong activity against the primary target in *Mycobacterium tuberculosis*, but also to be selective against the human off-targets, ensuring safe chronic oral administration. As shown in Figure 5, delamanid is inactive toward voltage-gated inwardly rectifying potassium channel KCNH2, also known as hERG (the human Ether-à-go-go-Related Gene), a major off-target in humans, with pIC_50_ below the detection limit. Unfortunately, its metabolite DM-6705 is a strong inhibitor of hERG, with an IC_50_ slightly below 100 nM (pIC_50_ = 7.09) which likely causes drug-induced QT prolongation associated with chronic use of delamanid.^31^ This example clearly illustrates that some drug metabolites may exhibit drug safety-related concerns in humans, thus requiring their close monitoring during the preclinical and clinical development of their parent molecules. The third example depicts a confirmed prodrug hydrocodone (third left) and its metabolically activated drug hydromorphone (third right), both acting as agonists of the kappa-type opioid receptor.^32^ While hydrocodone is a weak modulator of this receptor, as recorded by its binding affinity (*pK*_*i*_ = 6.59), its metabolite hydromorphone is nearly 100-fold more potent (*pK*_*i*_ = 8.55). This metabolic conversion (O-demethylation) is the topmost frequent reaction observed in the DM+ subset, and one of the reaction mechanisms preferentially found in this subset compared to its DM– counterpart (Table 1). The last example features indomethacin (bottom left), a nonsteroidal anti-inflammatory drug, alongside its inactive metabolite 5-hydroxyindomethacin (bottom right).^33^ This is a classic drug metabolism scenario: the parent drug is active against prostaglandin G/H synthase 2, but loses activity upon metabolism.

**Figure 5:**
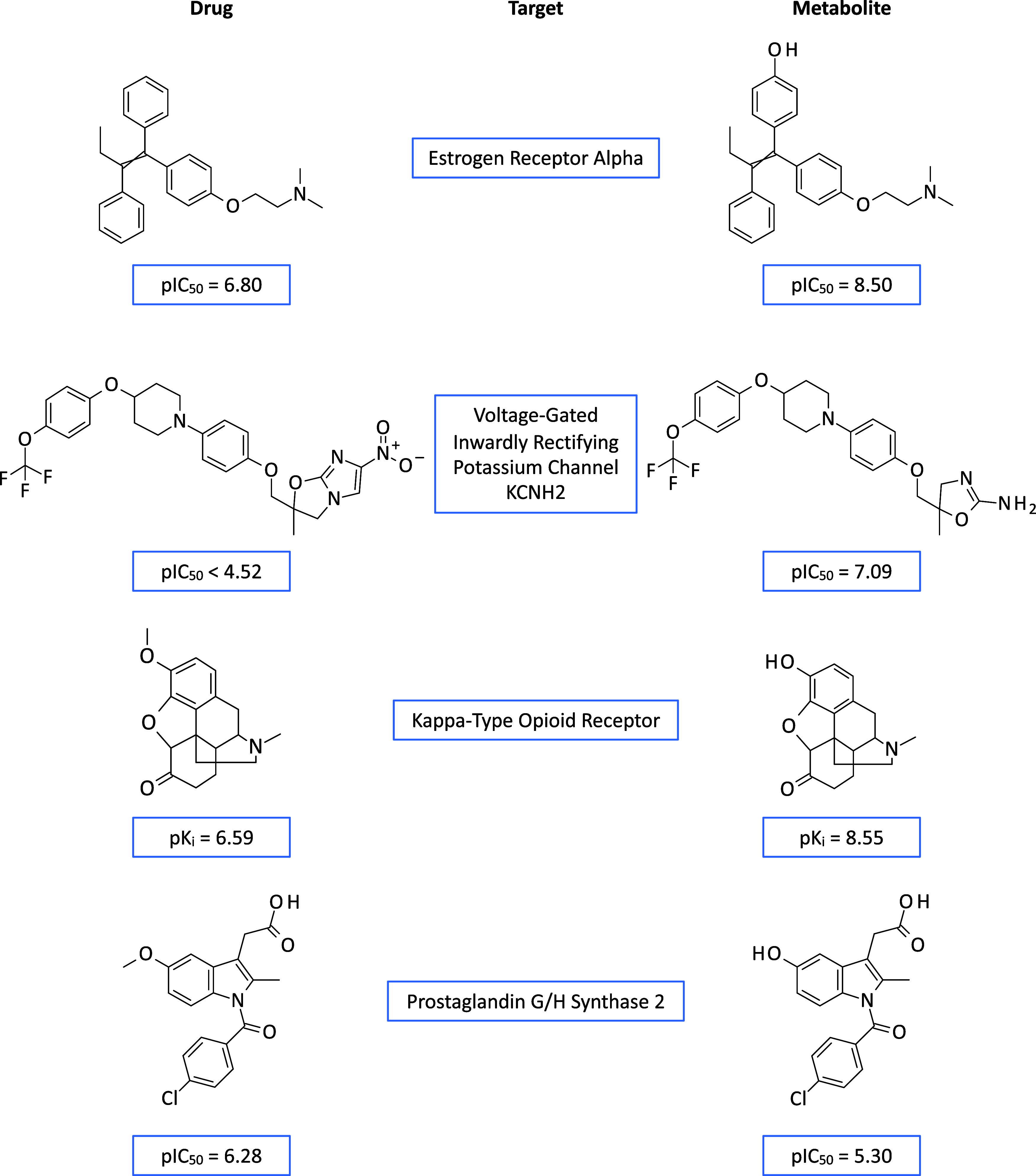
Four exemplary drug-metabolite pairs: tamoxifen and its active metabolite 4-hydroxytamoxifen, delamanid and its metabolite DM-6705 displaying strong off-target potential, a prodrug hydrocodone with its metabolically activated drug hydromorphone, and the drug indomethacin and its inactive metabolite 5-hydroxyindomethacin. Corresponding protein targets and respective bioactivity measurements for both the drug and the metabolite are shown. Note that stereochemistry has been removed during data curation.

## Conclusions

Here, we conducted a systematic computational analysis to identify drug-metabolite pairs where corresponding metabolites displayed retained or increased bioactivity compared to their parent drugs. For this, we started from a previously curated data set of drug-metabolite pairs (LAGOM) observed in humans, extended with drug-metabolite pairs related to top-selling drugs of 2018 (GLORYx), and related both drugs and metabolites to their bioactivity profiles recorded in the two major public databases (ChEMBL and BindingDB). The bioactivity annotations from these data sources were extracted and curated following well-defined, stringent criteria, and further aggregated and scrutinized to ensure agreement among the recorded bioactivity records coming from multiple data sources.

The resulting data set yielded 593 reaction-target pairs (i.e., drug-metabolite-target combinations), of which 155 (DM+ subset) were associated with a metabolite that had retained or increased bioactivity compared to its parent drug. In other words, 26% of all pairs were associated with active metabolites, which increased to 34% if only pairs with exact measurements were considered. Furthermore, 35% of all pairs had metabolites with activity equal to or lower than 100 nM, irrespective of the bioactivity profile of their parent drugs, which further increased the number of pairs with highly active metabolites. This demonstrates that drug metabolites frequently display high potency *in vitro*, which contradicts the common assumption that drug metabolites are less active or even inactive compared to their parent molecules. We also investigated whether these observations could be related to a high proportion of DM+ pairs containing prodrugs. However, only 9% of all DM+ pairs contained known prodrug-drug combinations, thus rendering the majority of pairs with detected active metabolites novel. It could also be hypothesized that *in vivo* outcomes of active metabolites may still be compromised due to typically inferior physicochemical and ADME properties. However, we observed no significant differences in calculated cLogP and TPSA, as well as the predicted LogD_7.4_ values of drugs and their metabolites (for both DM+ and DM−), suggesting that metabolites shared very similar lipophilicity and permeability profiles to their drug parents; HH CLint, nevertheless, was on average lower for metabolites. We also noted that certain transformation classes were more common for pairs containing active metabolites, such as O-demethylation and N-demethylation at a tertiary nitrogen. In addition, the membrane receptor target class was preferentially associated with pairs in the DM+ subset, as opposed to the enzyme target class that was more common in the DM– subset.

Our study shows that active metabolites of clinical drugs, based on the publicly disclosed bioactivity annotations of human targets, are more commonly observed than expected. This re-emphasizes that chemical compounds in preclinical and clinical development should be closely monitored for metabolites that may give rise to more potent on-target activity, safety concerns due to off-target engagement, or toxicity. Timely use of metabolite identification experiments, with focus on elucidating the actual metabolite structures, remains crucial in drug discovery. To support further research within this area, we make the drug-metabolite pairs of our full, curated data set publicly available as part of this work.

## Supplementary Material



## Data Availability

Code is available on GitHub at github.com/tsofiac/drug-metabolite-bioactivity. Data set is available on Zenodo at doi.org/10.5281/zenodo.18744003.

## Notes

Dictionary mapping the top ten reaction groups in the DM+ subset to their corresponding reaction template SMARTS (Table S1) (PDF)
