# Supplementary material for: Computational Identification of Active Drug Metabolites for Human Protein Targets
Source: Mol Pharm. 2026 Jun 18;23(7):3816–24. doi: 10.1021/acs.molpharmaceut.6c00258 (PMC13343518; doi:10.1021/acs.molpharmaceut.6c00258)
Supplement: Supplementary file 1 [file mp6c00258_si_001.pdf]

Supporting Information

Computational Identification of Active Drug Metabolites for Human Protein Targets

Sofia Larsson*[1,3], Rocío Mercado[1], Susanne Winiwarter[2], and Filip Miljković[3]

[1]Department of Computer Science and Engineering, Chalmers University of Technology and University of Gothenburg, Chalmersplatsen 1, 412 96, Gothenburg, Sweden
[2]Drug Metabolism and Pharmacokinetics, Research and Early Development, Cardiovascular, Renal and Metabolism (CVRM), BioPharmaceuticals R&D, AstraZeneca, Pepparedsleden 1, 431 83, Mölndal, Sweden
[3]Biopharma Chemistry, Discovery Sciences, BioPharmaceuticals R&D, AstraZeneca, Pepparedsleden 1, 431 83, Mölndal, Sweden

*Email: sofil@chalmers.se, sofia.larsson3@astrazeneca.com

Content

Table S1: Dictionary mapping the top ten reaction groups in the DM+ subset to their corresponding reaction template SMARTS.

Table S1: Dictionary mapping the top ten reaction groups in the DM+ subset to their corresponding reaction template SMARTS. The reaction group names are based on the most common reaction type observed with that reaction template in the dataset and do not guarantee that all instances of a reaction template correspond to the same reaction type.

Reaction Group	Reaction Template (SMARTS)
Deoxygenation (sulfoxide)	`[O-]-[S+;H0;D3:1]>>[S;H0;D2;+0:1]`
Conjugation (diverse)	`[OH;D1;+0:1]>>[O;H0;D2;+0:1]`
Hydroxylation (aliphatic chain)	`[CH3;D1;+0:1]>>[CH2;D2;+0:1]`
Hydroxylation (aliphatic ring)	`[CH2;D2;+0:1]>>[CH;D3;+0:1]`
Hydroxylation (aromatic)	`[cH;D2;+0:1]>>[c;H0;D3;+0:1]`
N-demethylation (sec. N)	`C-[NH;D2;+0:1]>>[NH2;D1;+0:1]`
N-demethylation (tert. N)	`C-[N;H0;D3;+0:1]>>[NH;D2;+0:1]`
O-dealkylation	`O-C-C-[O;H0;D2;+0:1]>>[OH;D1;+0:1]`
O-demethylation	`C-[O;H0;D2;+0:1]>>[OH;D1;+0:1]`
Oxidation (ketone)	`[CH;D3;+0:1]-[OH;D1;+0:2]>>[C;H0;D3;+0:1]=[O;H0;D1;+0:2]`
